# Boosting Performance of PANI@N‐WHL‐AC by a Modified Water‐in‐Salt‐Electrolyte Strategy for Zinc‐Ion Batteries

**DOI:** 10.1002/smll.74563

**Published:** 2026-07-14

**Authors:** Olbana Itana, Asmamew Taye, Xinwen Peng, Varsha Joseph, Amare Aregahegn, Reverant Crispin, Shimelis Admassie, Ziyauddin Khan

**Affiliations:** ^1^ Laboratory of Organic Electronics Department of Science and Technology (ITN) Linköping University Norrköping Sweden; ^2^ Center of Materials Science and Engineering Addis Ababa University Addis Ababa Ethiopia; ^3^ Department of Chemistry College of Natural and Computational Sciences Wallaga University Nekemte Ethiopia; ^4^ Department of Chemistry Asosa University Asosa Ethiopia; ^5^ State Key Laboratory of Pulp and Paper Engineering South China University of Technology Guangzhou China; ^6^ Wallenberg Wood Science Center Linköping University Norrköping Sweden; ^7^ Department of Chemistry Addis Ababa University Addis Ababa Ethiopia

**Keywords:** activated carbon, biomass, energy storage, PANI, water‐in‐salt electrolyte, zinc‐ion batteries

## Abstract

Aqueous zinc‐ion batteries (ZIBs) show promise for safe, affordable energy storage, but their performance is often constrained by the need for improved cathode design and electrolyte optimization to enable effective ion transport at interfaces. Here, a sustainable water hyacinth leaf‐derived N‐doped activated carbon/polyaniline composite (PANI@N‐WHL‐AC) is developed as a cathode for ZIBs and tested in an 8 m Zn(ClO_4_)_2_ electrolyte, regulated with methanesulfonic acid. The optimized electrolyte enhances reaction kinetics, reduces parasitic reactions, and stabilizes Zn^2+^ plating/stripping. As a result, the Zn||PANI@N‐WHL‐AC cell achieves a high specific capacity of approximately 105.6 mAh g^−1^ at 1.0 A g^−1^, an energy density of about 101.5 Wh kg^−1^, and a power density near 692.8 W kg^−1^, with approximately 80% capacity retention over 2500 cycles. This work highlights electrolyte‐managed dual‐ion kinetics as a valuable approach to unlocking the full potential of conducting polymer/biomass‐derived carbon composite cathodes in advanced aqueous ZIBs.

## Graphical abstract

MSA‐regulated water‐in‐salt electrolyte enables dual‐ion (Zn^2+^/H^+^) storage and stabilizes Zn plating/stripping in PANI@N‐doped biomass‐derived carbon cathodes. The synergistic electrolyte–electrode engineering delivers high energy density and long‐term cycling stability for aqueous zinc‐ion batteries.

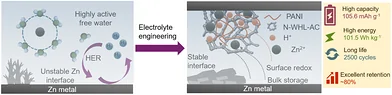

## 1 Introduction

Aqueous rechargeable zinc‐ion batteries (ZIBs) are attracting significant attention due to zinc's favorable redox potential (−0.76 V vs SHE), high theoretical capacity (820 mAh g^−1^), abundance, and environmental friendliness [1, 2, 3, 4]. The overall performance of ZIBs relies on the integrated functionality of the Zn anode, electrolyte, and cathode material. However, conventional cathode materials such as V‐based [5, 6,7], Mn‐based [8,9], layered double hydroxides [10, 11, 12] and Prussian blue analogues (PBAs) [13] often suffer from poor electrical conductivity, structural instability, and dissolution during repeated Zn^2+^ insertion/extraction cycles [13, 14, 15, 16, 17, 18].

Biomass‐derived carbon materials have emerged as promising sustainable and cost‐effective candidates for ZIB cathodes, offering hierarchical porosity, high surface area, and good electrical conductivity, which are the key features for enhancing electrochemical performance [17, 18, 19]. Among various biomass sources, water hyacinth (*Eichhornia Crassipes*), a fast‐growing and invasive aquatic plant prevalent in tropical and subtropical regions such as Africa and Southeast Asia, has garnered increasing interest due to its abundance, renewability, and low cost, making it a promising precursor for carbon material synthesis for energy storage applications [20, 21, 22]. While these materials exhibit capacitive charge storage behavior, high power density, and long‐lasting cycling stability, their energy density remains limited due to the dominant surface‐controlled mechanism, that favor supercapacitor‐like behavior rather than true battery operation. Further, N‐doping can enhance active sites and pseudocapacitance [23], but the fundamental limitation of insufficient faradaic contribution persists. To address this, redox‐active polymers such as polyaniline (PANI) have been integrated with carbon materials to introduce battery‐type charge storage. PANI offers a high theoretical capacity (∼295 mAh g^−1^ per electron), good electrical conductivity, fast redox kinetics, and a flexible conjugated structure that enables reversible Zn^2+^/H^+^ doping and de‐doping [24, 25, 26, 27, 28]. However, pure PANI suffers from several limitations, including low electroactive surface area, poor cyclic stability, a narrow redox potential range, and limited wettability in aqueous electrolytes [24, 25, 26, 27, 29, 30] which collectively hinder its performance in battery systems. Composites of PANI with biomass‐derived carbon have demonstrated synergistic performance, yet most studies rely on strong acidic electrolytes like H_2_SO_4_, which trigger electrode corrosion and degrade long‐term performance [31, 32, 33], in addition to the low energy density imposed by their narrow potential window (0.6 V) [33].

Electrolyte engineering provides a promising pathway to unlock the full potential of polyaniline@biomass‐derived activated carbon (PANI@AC) composite cathodes in ZIBs. Aqueous electrolytes are particularly attractive due to their intrinsic safety and ability to form efficient solvation sheaths around Zn^2+^ ions. However, despite their high ionic conductivity, they suffer from significant drawbacks, including zinc corrosion, hydrogen evolution, and dendrite formation [34, 35]. Moreover, the abundance of free water molecules accelerates the oxidative degradation of PANI at high cutoff potentials, where water attacks imine groups, triggering hydrolysis and compromising redox reversibility [36, 37, 38]. This degradation narrows the operating potential window and limits achievable energy density. In contrast, water‐in‐salt electrolytes (WiSEs) offer substantial advantages by addressing these limitations. WiSE formulations minimize free water content and promote the formation of a protective solid electrolyte interphase layer on the Zn anode, thereby suppressing parasitic reactions and extending the electrochemical stability window (ESW) [39, 40]. In this study, a WiSE based on 8 m Zn(ClO_4_)_2_ was employed to exploit the intrinsic advantages of highly concentrated aqueous electrolytes. Perchlorate (ClO_4_^−^) anions, classified as chaotropic species in the Hofmeister series, effectively disrupt the water‐water hydrogen‐bond network, thereby reducing free water activity and significantly widening the ESW [41, 42]. These characteristics make Zn(ClO_4_)_2_ (hereafter denoted as ZPC) based WiSE particularly attractive for achieving high energy density aqueous ZIBs. However, the highly concentrated electrolyte environment also introduces increased ion‐ion interactions and viscosity, which can adversely affect ionic transport and interfacial kinetics. Therefore, electrolyte composition optimization is essential to simultaneously balance ionic conductivity, interfacial stability, and electrochemical reversibility. To address this methanesulfonic acid (MSA) was introduced as a functional electrolyte additive to simultaneously regulate bulk electrolyte properties and electrode‐electrolyte interfacial behavior. The incorporation of MSA provides multiple synergistic benefits: (i) MSA is highly dissociated in aqueous media, effectively enhancing ionic conductivity by weakening excessive ion‐ion correlation in the concentrated electrolyte; (ii) its hydrophilic sulfonate functionality improves Zn^2+^/Zn interfacial reversibility and promotes more uniform Zn deposition behavior [43, 44, 45]; and (iii) the mobile –SO_3_H groups serve as a source of readily available protons, stabilizing the proton‐coupled redox transitions of PANI and improving electrochemical reversibility, performance and long‐term cycling stability. Through this electrolyte design strategy, the intrinsic advantages of ZPC‐based WiSE are retained while interfacial kinetics and charge transport are effectively optimized.

Motivated by these improvements, we demonstrate that the modified WiSE (8 m ZPC + 0.5 M MSA) is a highly effective electrolyte system for PANI@N‐doped water hyacinth‐derived activated carbon (PANI@N‐WHL‐AC) cathodes in ZIBs. The electrolyte modification simultaneously enhances ionic conductivity, improves the reversibility of Zn^2+^ plating/stripping, and enhances the redox activity of the PANI@N‐WHL‐AC cathode. As a result, the Zn||PANI@N‐WHL‐AC device delivers a high specific energy of 101.5 Wh kg^−1^, a specific power density of 692.8 W kg^−1^, and retains 80% capacity after 2500 cycles. These results highlight the strong synergy between rational cathode architecture and electrolyte design. Overall, this work presents a modified WiSE strategy that effectively overcomes key limitations of conventional aqueous electrolytes while significantly improving the electrochemical compatibility and performance of redox‐active organic molecule‐modified biomass‐derived porous carbon cathodes. These findings provide a viable pathway toward high‐energy, long‐cycle‐life, and sustainable ZIB systems.

## 2 Results and Discussion

### 2.1 Characterization of Materials

The surface properties of N‐WHL‐AC, PANI, and PANI@N‐WHL‐AC, including functional groups, surface morphology, and surface area, were systematically characterized using X‐ray photoelectron spectroscopy (XPS), scanning electron microscopy (SEM), and Brunauer‐Emmett‐Teller (BET) analysis, respectively. The XPS survey spectra (Figure S1 and Table S1) reveal distinct signals corresponding to C 1s, O 1s, and N 1s, confirming the presence of carbon, oxygen, and nitrogen elements in all samples. The successful incorporation of nitrogen into the carbon framework is attributed to the use of ammonium chloride as the nitrogen dopant precursor during synthesis. Nitrogen doping plays a crucial role in regulating the electronic structure and charge distribution within the carbon lattice of N‐doped WHL (N‐WHL‐AC). The introduction of nitrogen creates structural defects and alters the local electronic environment of carbon atoms, which can enhance electrical conductivity and electrochemical activity [46]. Notably, the XPS spectra of PANI@N‐WHL‐AC show significantly higher N and O signal intensities compared to N‐WHL‐AC, indicating the successful in situ polymerization of aniline on the activated carbon surface.

**FIGURE 1:**
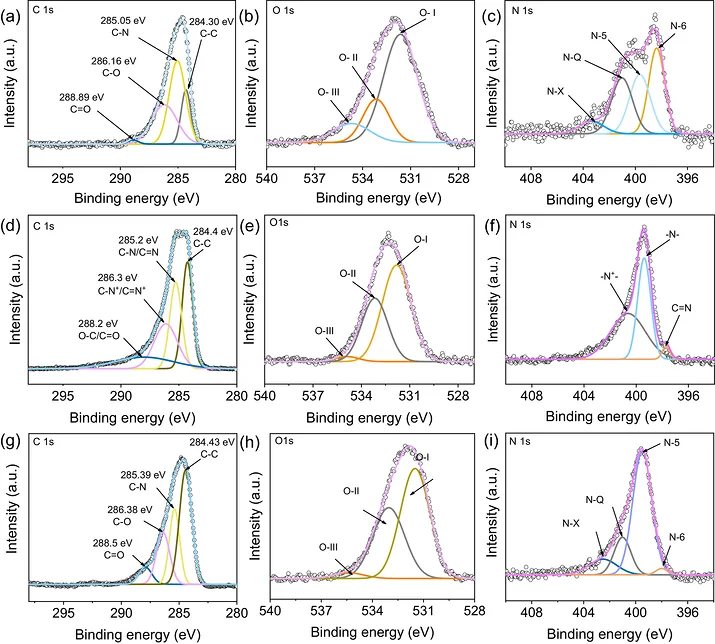
High‐resolution XPS spectra of N‐WHL‐AC (a–c), PANI (d–f), and PANI@N‐WHL‐AC (g–i), showing C 1s, O 1s, and N 1s, respectively. The spectra reveal the surface chemical states and functional groups associated with carbon, oxygen, and nitrogen species, confirming successful N‐doping of activated carbon and the effective incorporation of PANI onto the N‐WHL‐AC framework.

High‐resolution deconvolution of the XPS spectra provides further insight into the chemical bonding states. For N‐WHL‐AC, the C 1s spectrum (Figure 1a) is deconvoluted into four peaks at 284.30 eV, 285.05 eV, 286.16 eV, and 288.89 eV, corresponding to C═C, C─N, C─O, and C═O bonds, respectively. The O 1s spectrum (Figure 1b) consists of three components at 531.4 eV, 533.4 eV, and 535.5 eV, which are assigned to quinone‐type C═O (O‐I), phenolic/ether C─O (O‐II), and carboxylic COOH groups (O‐III). Similarly, the N 1s spectrum (Figure 1c) can be fitted into four sub‐peaks at 398.2, 399.7, 400.8, and 403.9 eV, corresponding to pyridinic nitrogen (N‐6), pyrrolic nitrogen (N‐5), graphitic nitrogen (N‐Q), and oxidized nitrogen (N‐X), respectively. Among these nitrogen functionalities, pyridinic‐N and pyrrolic‐N are known to enhance electrode wettability and contribute additional pseudocapacitive behavior through Faradaic redox reactions, particularly in acidic aqueous electrolytes. In contrast, graphitic‐N primarily improves electronic conductivity by substituting sp^2^‐hybridized carbon atoms and increasing the density of delocalized π‐electrons within the conjugated carbon framework [47, 48]. Overall, the observed C 1s, O 1s, and N 1s features of N‐WHL‐AC are consistent with previously reported results for N‐doped biomass‐derived carbons [49].

The high‐resolution XPS spectra of pristine PANI (Figure 1d–f) also agree well with literature reports [50]. The C 1s spectrum shows four characteristic peaks at 284.4 eV (C═C), 285.2 eV (C─N/C═N), 286.3 eV (C─N^+^/C═N^+^), and 288.2 eV (O─C/C═O). The N 1s spectrum exhibits three main peaks at ∼397.8, 399.6, and 400.6 eV, corresponding to pyridinic (C═N), amine (C─NH_2_), and pyridine N‐oxide species. Meanwhile, the O 1s spectrum shows peaks at 531.8, 533.19, and 535.0 eV, attributed to C═O (O‐I), C─O (O‐II), and adsorbed oxygen or moisture‐related species (O‐III), respectively. Further deconvolution of the PANI@N‐WHL‐AC high resolution spectra provides direct evidence of strong interfacial integration between PANI and the carbon framework (Figure 1g–i). The incorporation of PANI introduces a substantially higher nitrogen content, which enhances electrochemical performance through pseudocapacitive contributions, consistent with previous reports [51]. The C 1s spectrum shows four components at 284.43, 285.39, 286.38, and 288.5 eV, corresponding to C═C, C─N, C─O, and C═O bonds, respectively (Figure 1g). The prominent C─N bonding peak shifts from 285.2 eV for pristine PANI to 285.39 eV for PANI@N‐WHL‐AC, suggesting strong electronic interaction and successful integration of PANI on the activated carbon surface. This higher shift in binding energy likely reflects a decrease in electron density around the C─N bonds, which may arise from interfacial charge redistribution between PANI and the heteroatom‐rich N‐WHL‐AC framework. Such behavior can plausibly be associated with interfacial electronic interactions between PANI and the N‐doped activated carbon, where nitrogen functionalities and conjugated carbon domains may promote π‐π interactions and charge transfer, thereby modifying the local electronic structure of PANI. In addition, surface oxygen‐ and nitrogen‐containing groups could further influence the protonation state and local chemical environment of the imine (─C═N─) and amine (─NH─) units, contributing to the observed shift. These observations are consistent with previous reports on PANI–carbon composites and suggest strong electronic coupling at the interface [52, 53, 54, 55]. As shown in Figure 1h, the O 1s spectrum of PANI@N‐WHL‐AC consists of three peaks at 531.8 eV (C═O), 533.5 eV (C─O), and 535.5 eV (COOH/H─O─H). Meanwhile, the N 1s spectrum (Figure 1i) is deconvoluted into four peaks at 398.2, 399.7, 400.8, and 403.9 eV, corresponding to pyridinic‐N, pyrrolic‐N, graphitic‐N, and oxidized‐N, respectively [56]. These results collectively confirm the successful formation of a PANI@N‐doped biomass‐derived carbon composite with rich surface functionality and favorable electronic characteristics for zinc‐ion energy storage.

**FIGURE 2:**
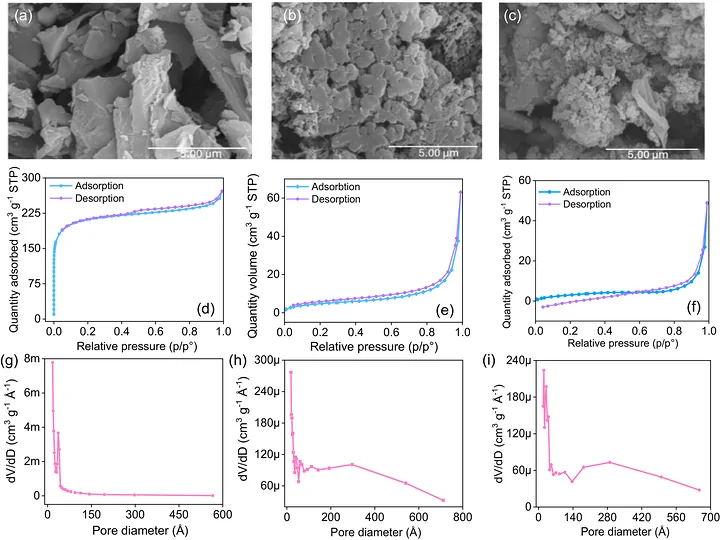
SEM images of (a) N‐WHL‐AC, (b) PANI, and (c) PANI@N‐WHL‐AC at 5 µm resolution. N_2_ adsorption/desorption isotherms of (d) N‐WHL‐AC, (e) PANI, and (f) PANI@N‐WHL‐AC. Panels (g–i) show the corresponding pore size distribution of N‐WHL‐AC, PANI, and PANI@N‐WHL‐AC, respectively.

The surface morphologies of PANI, N‐WHL‐AC, and PANI@N‐WHL‐AC were examined by SEM, as shown in Figure 2a–c. The N‐WHL‐AC sample exhibits a well‐developed and highly interconnected porous structure. This porosity originates from the ZnCl_2_ activation process, where metallic Zn intercalates into the carbon framework during activation and is subsequently removed during carbonization and post‐treatment, leaving behind abundant pore channels [57, 58]. Such interconnected porous networks facilitate rapid ion transport and act as effective electrolyte reservoirs, which are highly favorable for electrochemical energy storage. In contrast, the PANI electrode displays a relatively non‐uniform and rough surface morphology, consisting of interconnected nanospherical and oval‐like features, which are characteristic of polyaniline formed through oxidative polymerization. The SEM image of the PANI@N‐WHL‐AC composite reveals an irregular morphology, indicating successful deposition of PANI onto the activated carbon surface. The presence of PANI partially covers the carbon pores and induces some degree of particle agglomeration, which may lead to partial collapse or blockage of micropores. This morphological evolution is consistent with the reduction in porosity observed in the BET analysis.

To further investigate the pore structure, the as‐prepared materials (N‐WHL‐AC, PANI, and PANI@N‐WHL‐AC) were characterized by N_2_ adsorption‐desorption measurements, and the corresponding isotherms are presented in Figure 2d–f. The isotherms of PANI and PANI@N‐WHL‐AC resemble type III isotherms, displaying sharp adsorption behavior and indicating relatively broad pore‐size distributions with narrowed mesopores and widened micropores. The presence of a small hysteresis loop suggests capillary condensation, confirming the mesoporous nature of these materials which are considered as highways for ion transport [59]. In comparison, the N‐WHL‐AC sample exhibits an isotherm intermediate between type I and type IV, accompanied by an H4‐type hysteresis loop, indicative of a hierarchical porous structure composed of abundant micropores and mesopores, extending partially into the macropore region [60]. The H4 hysteresis loop observed in the relative pressure range of P/P_0_ = 0.5–1.0 is associated with capillary condensation within mesopores [61]. A slight adsorption upturn at high relative pressures (P/P0> 0.93) can be attributed to macropore filling or the aggregation of carbon frameworks within large pore cavities. The pore‐size distribution of N‐WHL‐AC, shown in Figure 2g confirms the dominance of micropores, accompanied by a smaller fraction of mesopores and macropores. Micropores (<2 nm) increase electroactive surface area and provide electrolyte‐ion adsorption sites, enabling charge accumulation and storage in electrical double layers. Mesopores (2–50 nm) hold ions and shorten the electrolyte diffusion paths, facilitating easier for Zn^2^^+^/H^+^ to move toward the interior active sites. Macropores (>50 nm) act as interconnected empty channels, increasing electrolyte penetration and reducing mass‐transfer resistance [62, 63]. Therefore, the hierarchical porous architecture of N‐WHL‐AC is expected to deliver high specific capacity and excellent rate performance in zinc‐based energy storage devices [64]. In contrast the PANI pore‐size distribution (Figure 2h) shows a limited microporous character with an extended pore distribution in the meso/macroprous region, implying relatively dense and aggregated polymer framework with limited N_2_‐accessible pore structure during BET measurement [65, 66]. The pore size distribution of PANI@N‐WHL‐AC (Figure 2i) displays remarkably reduced microporosity with a broader meso/macroporous interparticle‐void distribution. The specific surface area, pore volume, and average pore size of N‐WHL‐AC, PANI, and PANI@N‐WHL‐AC are summarized in Table 1. N‐WHL‐AC exhibits a high surface area owing to its rich microporous structure. However, the incorporation of PANI leads to a significant reduction in surface area, as the polymer occupies pore spaces and partially blocks the porous network, thereby decreasing the accessible surface area [67]. This structural evolution further supports the successful formation of the PANI@N‐WHL‐AC composite and explains the observed changes in electrochemical behavior.

**TABLE 1:** Specific surface area and pore volume measurements of N‐WHL‐AC, PANI, and PANI@N‐WHL‐AC using the BET and BJH methods, respectively.

Samples	BET Specific surface area (m^2^ g^−1^)	Average BJH Pore Volume (cm^3^ g^−1^)	Average BJH Pore size (nm)
N‐WHL‐AC	794.60	0.15	4.16
PANI	17.35	0.096	20.64
PANI@N‐WHL‐AC	12.27	0.075	20.07

### 2.2 Electrolytes Characterization

#### 2.2.1 ATR‐FTIR of Electrolytes

**FIGURE 3:**
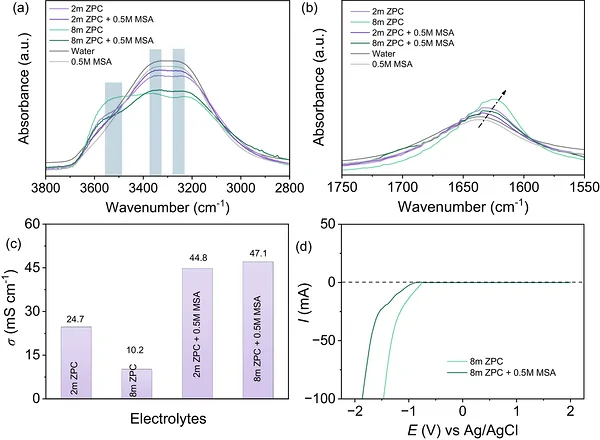
Physicochemical properties of electrolytes: (a, b) ATR‐FTIR spectra, (c) ionic conductivity measured by EIS, and (d) ESW determined using glassy carbon electrode (GCE), Ag/AgCl, and Pt mesh as the working, reference, and counter electrodes, respectively.

To gain molecular‐level insight into the solvation structure and hydrogen‐bonding environment of water molecules in various electrolyte systems, ATR‐FTIR spectroscopy was performed, as shown in Figure 3a,b and Figure S2a,b. The analysis primarily focuses on two characteristic vibrational bands of water: the broad O─H stretching mode centered around 3300 cm^−1^ and the H─O─H bending mode near 1640 cm^−1^. These bands are extremely sensitive to changes in the hydrogen‐bond network, making them effective indicators of solvation structure evolution. For baseline comparison, the ATR‐FTIR spectrum of 0.5 M MSA was measured as a control which displayed distinct peaks at 3300 cm^−1^ (O─H stretching), ∼1640 cm^−1^ (H─O─H bending), 1180 cm^−1^ (asymmetric stretching of ─SO_3_^−^), and 1050 cm^−1^ (symmetric stretching of ─SO_3_^−^), consistent with both water and sulfonate group vibrations.

The ATR‐FTIR spectra of 2 and 8 m ZPC electrolytes, with and without MSA, were subsequently analyzed. In the 2 m ZPC solution, the O─H stretching band showed a slight decrease in intensity, with no significant shift in band position, indicating limited alteration of the hydrogen‐bonding environment. However, in the 8 m ZPC system, a notable reduction in O─H stretching intensity was observed, accompanied by the appearance of a new band at ∼3550 cm^−1^, attributed to free water molecules weakly hydrogen‐bonded to perchlorate ions (OH···ClO_4_^−^) [68] (Figure 3a). This band emergence indicates a significant disruption of the water‐water hydrogen bond network, consistent with the chaotropic nature of ClO_4_^−^, which is known to disrupt the structured hydrogen‐bonding of bulk water [69, 70] despite its weak hydration properties. Furthermore, a broad, less intense band near 3230 cm^−1^ appeared in the 8 m ZPC spectrum. This feature is assigned to “ice‐like” water structures, where each water molecule forms four hydrogen bonds with its neighbors. In contrast, a broad, intense peak centered at ∼3370 cm^−1^ corresponds to “intermediate‐like” water structures, where water molecules are engaged in hydrogen bonding both with neighboring water molecules and ClO_4_^−^ ions (OH···OH···ClO_4_^−^) [40, 68, 71]. These results suggest the formation of a hybrid hydrogen‐bonding network, in which ClO_4_^−^ progressively replaces strong water‐water hydrogen bonds with weaker water‐anion interactions as its concentration increases, leading to broader and red‐shifted vibrational modes. The increasing peak intensity with red shift observed for the band between 1650‐1600 cm^−1^ as the concentration of electrolyte increases from 0 to 8 m can be attributed to the hydration‐ion pairs separation (Zn^2+^—(H_2_O)_n_—ClO_4_^−^) (Figure 3b). Within this wavenumber range, the spectra are overlapped with O‐H bending characteristic peaks of water molecules, showing that the perchlorate does not disrupt the water‐water molecules hydrogen bond structure in this wavenumber range. It can be inferred that the competition between the “structure breaking” affinity of perchlorate anions and the “structure making” affinity of Zn^2+^ cations, combined with the increasingly strong hydration shell of Zn^2+^ cations at higher concentration, is reflected in the red shift of the peaks [68].

Upon introduction of 0.5 M MSA into the 2 and 8 m ZPC solutions, a modest increase in O─H stretching band intensity was observed. This is attributed to increased free water content and the presence of additional –SO_3_H groups. However, the ClO_4_^−^ induced effects on the hydrogen‐bond network, particularly in the 8m solution, were not fully suppressed, confirming the persistence of WiSE behavior and the dominant influence of ClO_4_^−^ on the electrolyte's structural properties. Complementary information was derived from the fingerprint region of the ATR‐FTIR spectra (Figure S2a,b). Peaks at ∼1180 and ∼1050 cm^−1^ correspond to the asymmetric and symmetric stretching vibrations of the sulfonate (–SO_3_^−^) groups, respectively, originating from MSA [72, 73, 74]. These serve as reliable indicators of the presence and interaction of sulfonate moieties within the electrolyte matrix. In addition, several ClO_4_^−^ associated vibrational bands were identified: a strong asymmetric stretching mode near 1100 cm^−1^, a weaker symmetric stretching band at ∼930 cm^−1^, and a prominent bending vibration at ∼620 cm^−1^ [75, 76, 77, 78]. The weak band at ∼930 cm^−1^ is associated with infrared‐inactive or weakly active modes of free ClO_4_^−^, providing further evidence of uncoordinated perchlorate anions in the system. As the concentration of ClO_4_^−^ increases, either via salt concentration or MSA addition, both the 1100 and 620 cm^−1^ bands undergo a red shift (to lower wavenumbers) and increased intensity, indicative of the formation of solvent‐separated ion pairs or aggregated perchlorate species. This spectral evolution reflects a decrease in water coordination and the emergence of ion‐pairing and clustering phenomena at high salt concentrations.

Overall, these ATR‐FTIR results confirm that the 8 m ZPC electrolyte establishes a distinctly modified solvation environment, primarily due to the chaotropic disruption of water's hydrogen‐bond network by ClO_4_^−^ anions. The partial inclusion of MSA moderately alters this environment by introducing proton‐donating groups and increasing free water content but does not negate the hallmark features of a WiSE. These solvation structure changes are critical for understanding ion transport behavior, electrochemical stability, and interface compatibility in Zn‐based aqueous energy storage systems.

#### 2.2.2 Ionic Conductivity and ESW of Electrolytes

The 8 m ZPC electrolyte utilized in this study belongs to the WiSE class, defined by a high salt‐to‐solvent ratio. WiSEs are known to suppress side reactions, including HER, corrosion and dendritic growth while extending the ESW [79]. However, a major limitation of WiSE systems lies in their reduced ionic conductivity, which arises from increased viscosity and strong ion‐ion interactions due to the insufficient number of free water molecules available for solvation and ion mobility. To evaluate this trade‐off, the ionic conductivity (*σ*) of ZPC‐based electrolytes at both moderate and WiSE concentrations was measured, with and without MSA, using electrochemical impedance spectroscopy (EIS) (see Figure 3c). The measured ionic conductivities were 24.7 mS cm^−1^ for 2 m ZPC, 10.2 mS cm^−1^ for 8 m ZPC, 44.8 mS cm^−1^ for 2 m ZPC + 0.5 M MSA and 47.1 mS cm^−1^ for 8 m ZPC + 0.5 M MSA. These results reveal a significant decline in conductivity as the salt concentration increases from 2 to 8 m, consistent with the viscosity‐limited ion mobility and intensified ion pairing or clustering commonly observed in highly concentrated electrolytes. Remarkably, the introduction of 0.5 M MSA into both the 2 and 8 m ZPC systems led to a substantial improvement in ionic conductivity, particularly in the WiSE regime. This enhancement is attributed to the high degree of dissociation of MSA in aqueous media. As a strong, fully ionized organic acid, MSA promotes increased ion dissociation throughout the electrolyte, thereby reducing ion–ion correlations and enhancing ion transport. In the case of 8 m ZPC, the ionic conductivity improved from 10.2 to 47.1 mS cm^−1^ upon MSA addition, highlighting the dual role of MSA in both modifying the solvation environment and mitigating transport limitations in concentrated electrolyte systems. These findings demonstrate that electrolyte tailoring using proton‐donating additives like MSA can serve as an effective strategy to overcome conductivity challenges in WiSE systems, paving the way for their practical use in high‐energy aqueous ZIBs.

The ESW of the electrolyte was evaluated using linear sweep voltammetry in a three‐electrode configuration at a scan rate of 0.5 mV s^−1^. As shown in Figure 3d, the 8 m ZPC electrolyte without MSA exhibited a cathodic onset potential of approximately −0.76 V vs Ag/AgCl, corresponding to the initiation of the HER and/or Zn deposition. Interestingly, upon the addition of 0.5 M MSA, this cathodic onset shifted to −0.96 V vs Ag/AgCl (more negative), indicating a marked extension of the ESW cathodically. On the anodic side, both electrolytes showed similar behavior, with a gradual increase in current density and no significant difference in oxidative stability. The cathodic stability limit was shifted toward more negative potentials upon the addition of MSA. This enhancement can be rationalized by considering the strong interactions between MSA and water molecules [80]. MSA can form hydrogen‐bonded structures and ion‐associated –CH_3_SO_3_^−^/H_3_O^+^ clusters, particularly under conditions of reduced hydration. These interactions reorganize the hydrogen‐bond network of water and decrease the fraction of bulk‐like “free” water that is readily available to participate in the HER [40]. Such restructuring effectively lowers water activity and delays the onset of HER, thereby extending the cathodic stability limit. This interpretation is consistent with broader observations in WiSE and structurally modified aqueous electrolytes, where reduced water activity and altered interfacial water structure correlate strongly with improved electrochemical stability. In highly concentrated mixed‐anion systems such as the present electrolyte, it may also suggest that methanesulfonate anions may interact with Zn^2+^ ions, either through partial coordination or by influencing the second solvation shell. Such modifications of the Zn^2+^ solvation environment could indirectly tune interfacial kinetics and deposition behavior [81]. While this remains a mechanistic hypothesis, it is consistent with established principles governing solvation chemistry in advanced aqueous zinc electrolytes. Taken together, the combined effects of water‐structure reorganization and potential Zn^2+^ solvation tuning provide a coherent explanation for the suppressed HER activity and the more reversible Zn plating/stripping behavior observed experimentally. The improved cycling stability and smoother Zn deposition morphology further support this interpretation. Therefore, the role of MSA in the ZPC‐based electrolyte extends beyond simple proton donation; it functions as a regulator of both water structure and Zn^2+^ solvation chemistry. The mechanistic discussion presented here reflects a plausible interpretation grounded in current understanding of concentrated aqueous electrolyte systems.

### 2.3 Zn Anode Performance in Electrolyte

**FIGURE 4:**
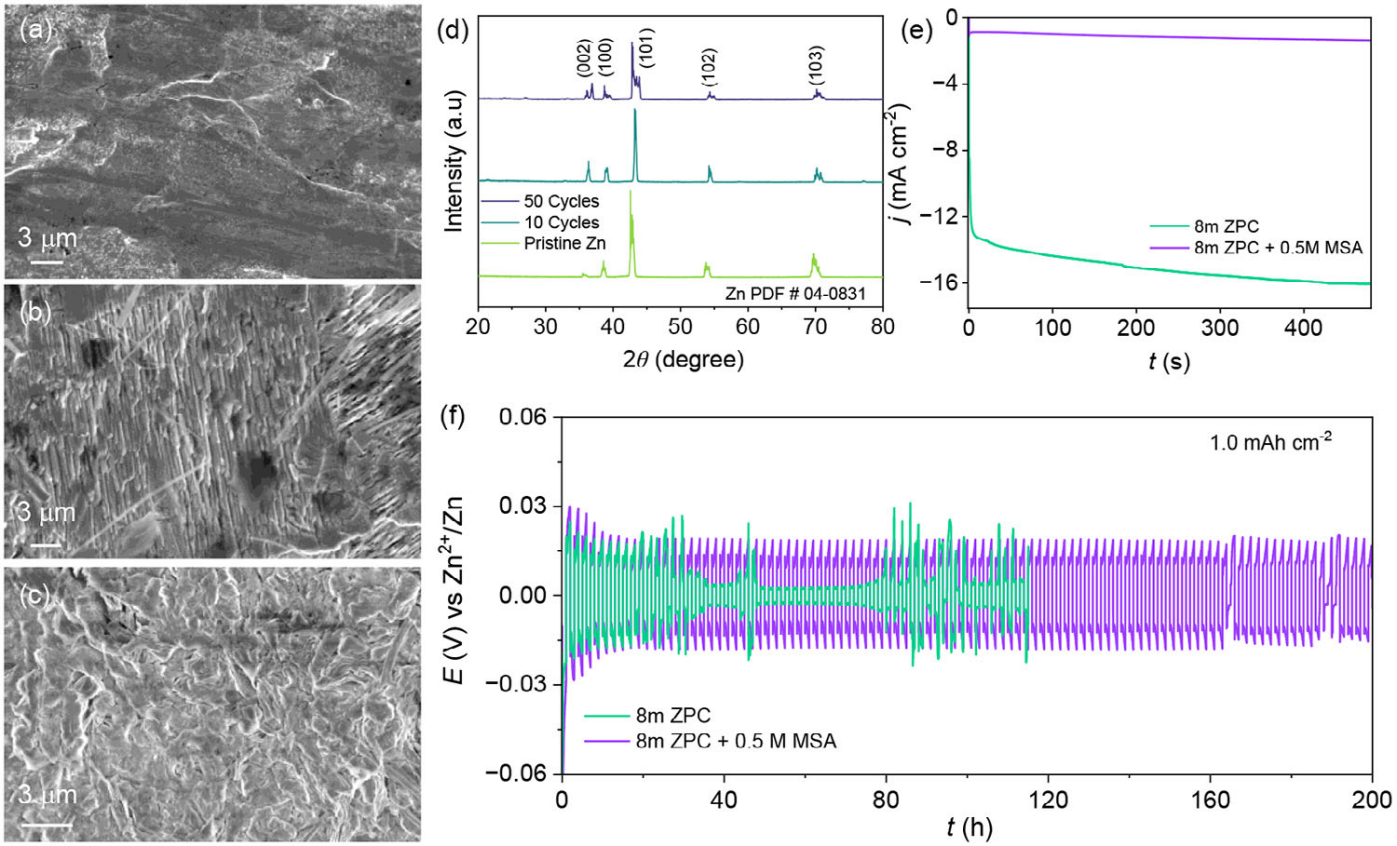
SEM images of Zn foil: (a) pristine and after Zn deposition following GCD cycling in 8 m ZPC + 0.5 M MSA after (b) 10 cycles and (c) 50 cycles. (d) XRD patterns of deposited Zn after 10 cycles and 50 cycles in 8m ZPC + 0.5 M MSA. (e) CA at ‐150 mV of Zn||Zn symmetric cell and (f) Zn^2+^ plating/stripping at 1 mAh cm^−2^.

In ZIBs, the plating and stripping of Zn at the anode is a critical half‐cell reaction that directly influences battery performance and longevity. Therefore, investigating the Zn deposition behavior in the proposed electrolyte is essential. To probe Zn plating/stripping kinetics and interfacial stability, CV measurements were performed in Zn||Zn symmetric cells at scan rates ranging from 1 to 10 mV s^−1^ (Figure S3). In the pristine 8 m ZPC electrolyte (Figure S3a), the low‐scan‐rate CV curves exhibit distinct Zn deposition and stripping features, including a nucleation‐related crossover characteristic of Zn electrochemistry. However, these features become progressively distorted at higher scan rates, accompanied by increased polarization and reduced reversibility, suggesting sluggish interfacial kinetics [82]. In contrast, the addition of 0.5 M MSA results in more symmetric and reproducible CV profiles across the investigated scan‐rate range, with reduced current overshoot and improved overlap between successive scans (Figure S3b). These observations indicate enhanced Zn plating/stripping reversibility and more stable interfacial kinetics in the MSA‐containing electrolyte. The improved electrochemical response is consistent with the modified solvation environment and interfacial interactions introduced by MSA, which facilitate more uniform Zn deposition behavior and improved Zn^2+^ transport. Further, the Zn plating/stripping process was studied using SEM and XRD analyses of Zn anodes retrieved from symmetric Zn||Zn symmetric cells. Two symmetric cells were assembled and cycled galvanostatically in 8 m ZPC + 0.5 M MSA for 10 and 50 plating/stripping cycles respectively, each at an areal capacity of 1mAh cm^−2^. As a reference, pristine Zn foil (before cycling) was also characterized for comparison. As shown in Figure 4a, the surface of the pristine Zn anode appears relatively smooth, compact, and uniform, with only minor polishing marks visible, indicative of a clean and dense metallic surface prior to any electrochemical reaction. After 10 cycles, as shown in Figure 4b, the Zn surface develops well‐aligned compact columnar structures, suggesting that the 8 m ZPC + 0.5 M MSA electrolyte enables uniform zinc nucleation and growth, while effectively suppressing dendrite formation during the initial plating/stripping cycles. Upon increasing the cycle number to 50 (Figure 4c), the Zn surface evolves into a flake‐like, yet still compact morphology. This indicates a degree of structural evolution over extended cycling, but importantly, there is no apparent formation of dendritic structures, even after prolonged cycling. These results confirm that MSA‐modified WiSE not only supports reversible Zn deposition but also promotes surface stability and mitigates dendrite growth.

To further assess the phase purity and crystallographic orientation of the deposited Zn, XRD was performed on Zn foils obtained from symmetric Zn||Zn cells after galvanostatic cycling in 8 m ZPC + 0.5 M MSA electrolyte. The XRD patterns of pristine Zn foil and Zn electrodes after 10 and 50 plating/stripping cycles are presented in Figure 4d. After 10 cycles, the XRD pattern exhibits well‐defined diffraction peaks corresponding to (002), (100), (101), (102), and (103) planes of metallic Zn. Among these, the (002) plane shows a pronounced intensity, indicating a preferential growth along the (002) direction. This observation aligns well with prior studies suggesting that Zn deposition along the (002) plane promotes uniform nucleation and suppresses dendritic growth and other aqueous‐electrolyte related instabilities at the Zn surface [83, 84, 85, 86]. These observations are consistent with SEM results (Figure 4b) where compact and well‐aligned columnar structures were observed after 10 cycles, supporting the notion of controlled, oriented Zn growth in MSA‐modified electrolyte. When the number of cycles increased to 50, the XRD pattern of the Zn electrode revealed twin‐like splitting along the entire crystallographic planes. This peak splitting has been attributed to textural changes in Zn during repeated Zn^2+^ plating/stripping processes, rather than the formation of new crystalline phases [87, 88]. Furthermore, in comparison to pristine Zn, the diffraction peaks after both 10 and 50 cycles showed a slight shift toward higher 2*θ* values. This shift suggests a reduction in interplanar spacing likely due to compressive residual stress, which can develop from cyclic mechanical deformation of the Zn surface during repeated plating/stripping. Noticeably, there are no additional diffraction peaks at low angles (<30 °) or in the 34–36 ° region that would typically be associated with corrosion byproducts such as Zn(OH)_2_ or ZnO. These products are common indicators of Zn anode degradation in aqueous systems and generate distinct signatures in XRD patterns [89]. The absence of such peaks, combined with the compact and no obvious dendritic observed in the SEM images after 50 (Figure 4c), confirms that corrosion and surface passivation are effectively suppressed in 8 m ZPC + 0.5 M MSA electrolyte. The improved Zn reversibility observed in the MSA‐containing electrolyte may also be associated with modifications of the interfacial proton environment near the Zn surface. The addition of MSA is expected to influence proton activity and the local solvation structure, which could contribute to more uniform Zn deposition and reduced parasitic reactions. Although direct measurement of the local pH at the Zn/electrolyte interface was not performed in this study, such measurements are experimentally challenging because the interfacial pH evolves dynamically during Zn plating/stripping and generally require specialized operando techniques. Therefore, the proposed role of MSA on the interfacial proton environment should be regarded as a plausible mechanistic interpretation consistent with observed electrochemical behavior.

The long‐term Zn plating/stripping behavior in both 8 m ZPC and 8 m ZPC + 0.5 M MSA electrolytes was further investigated under galvanostatic conditions at a fixed areal capacity of 1 mAh cm^−2^. As shown in Figure 4f, the Zn||Zn symmetric cell using 8 m ZPC + 0.5 M MSA exhibited stable and uniform voltage polarization for over 200 h, indicating highly reversible Zn plating/stripping. In contrast, the cell using pristine 8 m ZPC displayed pronounced voltage fluctuations and instability, leading to short‐circuit failure within 30 h of cycling. Consistent with the electrochemical behavior, post‐cycling SEM images of Zn electrodes recovered from the symmetric cells (Figure S4) reveal a more compact and uniform Zn surface morphology in the MSA‐containing electrolyte, whereas the Zn electrode cycled in pristine 8 m ZPC exhibits a heterogeneous and cracked surface, indicative of non‐uniform Zn deposition and repeated surface reconstruction during cycling. To further evaluate the chemical stability of Zn in the two electrolytes, Zn foils were immersed in 8 m ZPC and 8 m ZPC + 0.5 M MSA for 72 h, followed by XRD analysis (Figure S5). The XRD patterns were dominated by the characteristic diffraction peaks of metallic Zn indicating that prolonged electrolyte exposure does not induce significant crystalline corrosion products. Nevertheless, no major phase transformation of the Zn electrode was detected. These results, together with the electrochemical cycling behavior, indicate that the addition of MSA improves interfacial stability and promotes more uniform Zn deposition/dissolution. Consequently, the MSA‐containing electrolyte effectively mitigates the instability commonly associated with prolonged Zn cycling, whereas the rapid failure observed in pristine 8 m ZPC is consistent with non‐uniform Zn deposition and progressive electrode degradation. To further investigate Zn deposition behavior, chronoamperometry (CA) measurements were conducted at a fixed potential of −150 mV vs Zn^2+^/Zn, as shown in Figure 4e. In the 8 m ZPC electrolyte, the deposition current density continued to increase for over 450 s, suggesting 3D diffusion behavior due to uneven and localized Zn deposits. This is typically associated with uncontrolled nucleation, leading to rough and dendritic Zn growth. In contrast, the CA response in 8 m ZPC + 0.5 M MSA displayed relatively steady current density, indicative of 2D diffusion‐dominated growth. This behavior reflects a more controlled and homogenous Zn deposition process, further supporting the role of MSA in regulating the Zn^2+^ reduction kinetics and promoting smooth, dendrite‐free Zn layers. Further, the Zn deposition reversibility was evaluated using a Ti||Zn asymmetric cell in the 8 m ZPC + 0.5 M MSA electrolyte. Typically, an asymmetric Ti||Zn configuration is employed to determine the coulombic efficiency (CE) of Zn deposition/stripping, as the Ti electrode contains no initial zinc and therefore serves as an ideal inert substrate for assessing Zn plating reversibility. As shown in Figure S6, the initial coulombic efficiency was approximately 90%, which gradually increased with cycling and reached >99% after 50 cycles. This progressive improvement indicates stabilization of the Zn deposition/stripping process. For comparison, the Ti||Zn cell in pristine 8 m ZPC exhibited a lower average CE of ∼93% over the same cycling period, whereas the MSA‐containing electrolyte maintained an average CE exceeding 99%, highlighting the beneficial effect of MSA on Zn plating/stripping reversibility. Importantly, the high and stable CE demonstrates that the majority of Zn deposited on the Ti substrate can be effectively stripped during subsequent stripping cycles, confirming the excellent reversibility of Zn plating in the MSA‐modified electrolyte.

### 2.4 Electrochemical Performance of Devices

#### 2.4.1 PANI@N‐WHL‐AC

**FIGURE 5:**
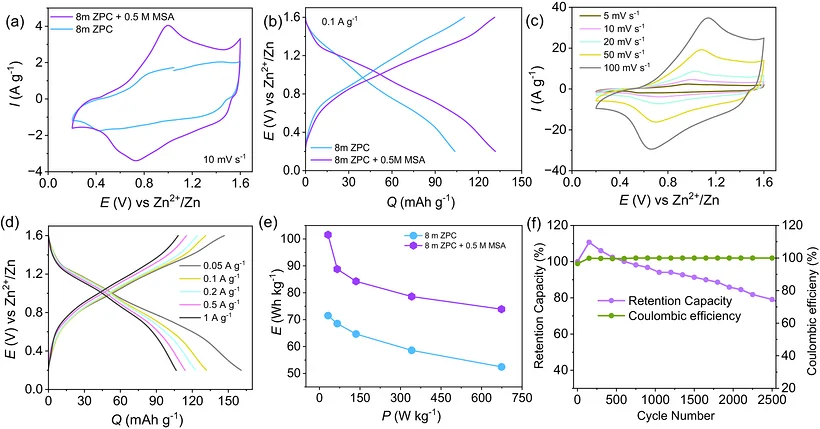
(a) CV at 10 mV s^−1^ and (b) GCD at 0.1 A g^−1^ of Zn||PANI@N‐WHL‐AC in 8 m ZPC and 8 m ZPC + 0.5 M MSA. (c) CV and (d) GCD of PANI@N‐WHL‐AC in 8 m ZPC + 0.5 M MSA at different rates. (e) Ragone plot. (f) Cyclic stability and coulombic efficiency of PANI@N‐WHL‐AC in 8 m ZPC + 0.5 M MSA at 1.0 A g^−1^.

To systematically evaluate the electrochemical behavior of the PANI@N‐WHL‐AC composite, CV and GCD measurements were performed within a potential window of 0.2–1.6 V using a two‐electrode configuration in both 8 m ZPC and 8 m ZPC + 0.5 M MSA electrolytes. The upper cutoff voltage was limited to 1.6 V because extending the charging potential beyond this value has been reported to induce irreversible over‐oxidation of polyaniline, leading to the formation of benzoquinone‐type species and consequent capacity fading [38]. This voltage window, therefore, ensures electrochemical stability while enabling reversible redox activity of PANI. As shown in Figure 5a,b, both the CV response and discharge capacity of PANI@N‐WHL‐AC electrode are significantly enhanced in MSA‐containing electrolyte compared to the MSA‐free system. This improvement in electrochemical performance is primarily attributed to the presence of protons (H^+^), which facilitate reversible proton‐coupled redox reactions along the PANI backbone. Protons act as additional charge carriers in the dual‐ion storage system, thereby enhancing both energy storage and interfacial charge‐transfer kinetics. In addition to Zn^2+^ ions, H^+^ ions in mildly acidic aqueous electrolytes have recently gained considerable attention for their role in Zn‐organic battery systems, owing to their ultrafast insertion/extraction behavior [90, 91]. The superior kinetic behavior of H^+^ originates from its exceptionally high ionic mobility, smaller effective hydrated radius (∼0.1 nm), and weaker solvation energy, compared to Zn^2+^, which has a larger hydrated radius (∼0.43 nm) and much stronger solvation energy [92, 93, 94]. As a result, H^+^ ions preferentially participate in the charge storage process by interacting with the imine (─C═N) moieties of π‐conjugated PANI backbone, enhancing both redox reversibility and rate performance in aqueous ZIBs.

As a control, the electrochemical performance of N‐WHL‐AC and PANI was also evaluated in 8 m ZPC + 0.5 M MSA electrolyte, as shown in Figure S7. The CV profiles N‐WHL‐AC and PANI electrodes recorded at 10 mV s^−1^ in 8 m ZPC + 0.5 M MSA electrolyte exhibited distinct electrochemical signatures that reflect their respective charge storage mechanisms. The quasi‐rectangular CV shape of N‐WHL‐AC indicates that its charge storage is primarily governed by electrical double layer capacitance (EDCL), which arises from reversible ion adsorption/desorption of Zn^2+^ ions at the electrode‐electrolyte interface, a feature commonly reported for biomass‐derived porous carbons used in aqueous zinc‐based energy storage systems. In contrast, the CV profile of the PANI electrode displays well‐defined and reversible redox peaks, corresponding to its characteristic faradaic redox reaction transitions along the conjugated polymer backbone, confirming the pseudocapacitive nature of PANI, which contributes to energy storage through fast redox reactions involving both Zn^2+^ and H^+^ ion interactions with the polymer chain. In contrast to these two, the PANI@N‐WHL‐AC composite exhibits a broadened, hybrid CV shape that integrates features from both EDLC and pseudocapacitive mechanisms. The CV curve combines the capacitive characteristics of the carbon framework with the redox activity of PANI, indicating synergistic charge storage behavior. The increased enclosed area of the CV curves suggests enhanced overall charge storage capacity, where the redox‐active PANI provides faradaic contributions, while the high surface area biomass‐derived activated carbon facilitates rapid ion transport and contributes to improved rate capability.

The charge‐discharge performance of N‐WHL‐AC and pristine PANI electrodes was evaluated under identical conditions, and the results are presented in Figure S7b,d. The N‐WHL‐AC electrode exhibits relatively symmetric charge–discharge profiles, delivering a specific capacity of 48.4 mAh g^−1^ at 0.1 A g^−1^ (Figure S7b). This behavior is characteristic of a predominantly capacitive charge storage mechanism, governed by reversible ion adsorption and desorption at the electrode/electrolyte interface. In contrast, the pristine PANI electrode displays sloping discharge profiles with a lower specific capacity of 43.6 mAh g^−1^ at 0.1 A g^−1^ (Figure S7d). The comparatively limited performance can be attributed to the restricted accessibility of electroactive sites and sluggish ion diffusion within the bulk PANI matrix, despite its intrinsically high electronic conductivity [95, 96, 97]. These kinetic limitations hinder the full utilization of PANI's redox‐active backbone. By comparison, the PANI@N‐WHL‐AC composite demonstrates significantly enhanced electrochemical performance, as evidenced by extended discharge profiles and well‐defined voltage plateaus indicative of improved faradaic activity. The composite delivers a higher specific capacity of 132 mAh g^−1^ at 0.1 A g^−1^, outperforming both individual components. This improvement highlights the synergistic integration of PANI with N‐WHL‐AC, where the porous carbon scaffold provides a high surface area, efficient electron transport pathways, and shortened ion‐diffusion distances, while PANI contributes additional pseudocapacitive and faradaic redox activity through its conjugated structure.

Further electrochemical evaluation of the PANI@N‐WHL‐AC electrode was conducted using CV and GCD measurements in 8 m ZPC + 0.5 M MSA electrolyte in the same potential window, as shown in Figure 5c,d. The CV curves recorded at various scan rates (Figure 5c) exhibit a proportional increase in current response with increasing scan rate, indicating favorable charge‐transfer kinetics and efficient ion transport at the electrode/electrolyte interface. Importantly, the redox features remain well defined without significant distortion, suggesting good electrochemical reversibility. The corresponding GCD profiles (Figure 5d) display distinct and stable discharge plateaus across different current densities, further confirming the electrode's robust redox behavior and excellent rate capability. The PANI@N‐WHL‐AC electrode delivers a maximum specific capacity of 160 mAh g^−1^ (calculated based on the composite cathode mass, excluding the Zn foil) at a current density of 50 mA g^−1^. Upon increasing the current density to 1000 mA g^−1^, the specific capacity decreases to 107 mAh g^−1^, demonstrating good capacity retention under high‐rate conditions. The observed decrease in specific capacity at higher scan rates and current densities can be attributed to kinetic limitations associated with ion diffusion and migration within the electrode matrix. At lower current densities, electrolyte ions have sufficient time to penetrate deeper into the electroactive material, enabling more complete utilization of active sites. In contrast, at higher scan rates, the diffusion process becomes rate‐limiting, restricting ion access primarily to surface or near‐surface regions, thereby reducing capacity.

As summarized in Figure 5e (Ragone plot), the PANI@N‐WHL‐AC electrode delivers a specific capacity of 105.6 mAh g^−1^ at 1 A g^−1^, corresponding to a specific energy density of 101.5 Wh kg^−1^ and a specific power density of 692.8 W kg^−1^ in the MSA‐containing electrolyte. In comparison, the electrode achieves 77.6 mAh g^−1^ of specific capacity, 71.5 Wh kg^−1^ of specific energy, and 674.1 W kg^−1^ of specific power, respectively, in the 8 m ZPC electrolyte. The enhanced energy and power output in the presence of MSA demonstrate the beneficial role of electrolyte modification in improving transport kinetics and redox reversibility. Notably, the substantial increase in energy density highlights the effectiveness of MSA in facilitating deeper and more reversible charge storage. Given that energy density is a critical parameter for practical applications, including portable electronics, electric vehicles, and grid‐scale energy storage, the improved Ragone performance underlines the potential of the ZPC‐based electrolyte system for advanced ZIBs.

The long‐term cycling performance of the Zn||PANI@N‐WHL‐AC cell was evaluated in 8 m ZPC + 0.5 M MSA, and the results are presented in Figure 5f. The device exhibits a capacity retention of 80% with a nearly 100% coulombic efficiency after 2500 cycles at 1.0 A g^−1^, demonstrating highly reversible Zn plating/stripping behavior and sustained proton‐coupled redox activity within the PANI backbone. The capacity fading commonly observed in PANI‐based systems, which is often attributed to insufficient proton availability and susceptibility to hydrolysis at high cutoff voltages in aqueous electrolytes [24], is effectively mitigated by the incorporation of MSA, as evidenced by the stable long‐term cycling performance. MSA performs a dual functional role in the electrolyte system. First, it acts as a proton donor, stabilizing the imine (─C═N─) groups of PANI and facilitating reversible proton‐coupled redox transitions between the emeraldine base and emeraldine salt forms. This stabilization enhances capacity retention and improves redox kinetics, thereby enabling stable and prolonged charge‐discharge cycling in the ZIB. Second, the mesylate anion (CH_3_SO_3_^−^), owing to its relatively bulky structure and strong interfacial affinity, may adsorb onto the Zn anode surface, promoting more uniform Zn deposition and suppressing dendrite formation. This interfacial stabilization is further supported by Zn||Zn symmetric cell measurements, where the plating/stripping lifetime was significantly extended from approximately 30 h (without MSA) to 200 h in the presence of MSA. These electrochemical observations collectively indicate that significant structural degradation of PANI is effectively suppressed under the MSA‐modified electrolyte conditions.

Self‐discharge, defined as the spontaneous decay of voltage or capacity in a charged device under open‐circuit conditions, remains a significant challenge in aqueous organic battery systems. In many cases, the issue is so pronounced that it is either insufficiently reported or not systematically evaluated in literature. The self‐discharge behavior of the Zn||PANI@N‐WHL‐AC device was investigated by first charging the cell for five cycles to 1.6 V at a current density of 0.5 A g^−^^1^, followed by chronoamperometric polarization at 1.6 V for 2 h to ensure equilibration. Subsequently, the cell was allowed to rest under open‐circuit voltage (OCV) conditions for 120 h at room temperature, during which the voltage decay was monitored. As shown in Figure S8a, the cell in the 8 m ZPC + 0.5 M MSA electrolyte exhibited a gradual voltage decay from 1.6 V to 1.1 V over 120 h and retained approximately 69% of its initial voltage, corresponding to a suppressed voltage decay rate of 4.2 mV h^−1^. In comparison, the cell using pristine 8 m ZPC showed a slightly higher voltage decay rate of approximately 5 mV h^−1^ under identical conditions, indicating that the addition of MSA moderately suppresses self‐discharge. Furthermore, the remaining capacity after OCV aging was evaluated by discharging the cell immediately after the 120 h rest period. As shown in Figure S8b, the device in the 8 m ZPC + 0.5 M MSA electrolyte retained 53.6% of its initial capacity, corresponding to a capacity loss of 46.4% after prolonged rest. This behavior indicates a moderate self‐discharge rate, which is commonly observed in pseudocapacitive and redox‐active aqueous zinc energy storage systems due to the presence of soluble redox species and fast interfacial reactions.

The overall electrochemical performance of the developed materials is summarized and compared with previously reported studies in Table 2 and Table S2 (which provide comparative information on self‐discharge in PANI‐, carbon‐, and other material‐based Zn systems), highlighting the competitive energy density, cycling stability, and rate capability achieved in this work.

**TABLE 2:** PANI@biomass derived AC for energy storage from the literature.

Electrode	Electrolyte	Specific Capacitance/capacity	Energy density, Power density	capacitance Retention	Cycle number	Refs.
PANI@AC	1 M H_2_SO_4_	214 F g^− 1^ at 1.0 A g^− 1^	—	94.55%	5000	[98]
PANI@N, S‐AC	1 M H_2_SO_4_	304 F g^− 1^ at 1 A g^− 1^	23.6 Wh kg^−1^ ; 395 W kg^− 1^	90.2%	5000	[99]
PANI@N‐AC	1 M H_2_SO_4_	347 F g^−^^1^ at 2 A g^−^^1^	44.4 Wh kg^−^^1^; 922 W kg^−^^1^	99%	2500	[67]
PANI@CS*	1.0 M H_2_SO_4_	285 F g^−1^ at 0.5 A g^−1^	22.2 Wh kg^−1;^ 713 W kg^−1^	80%	5000	[100]
PANI@YC*	1.0 M H_2_SO_4_	100 F g^−1^ at 1 A g^−1^	16.9 Wh kg^−1^; 550 W kg^−1^	95.4%	5000	[101]
PANI@N‐WHL‐AC	8m ZPC +0.5 M MSA	105.6 mAh g^−1^ at 1 A g^−1^	101.5Whkg^−1^; 692.8 W kg^−1^	80%	2500	This work

CS‐self‐doped carbon, YC‐yeast derived N‐doped carbon, F g^−1^‐ for supercapacitor type device, mAh g^−1^‐ for battery type device.

#### 2.4.2 Energy Storage Mechanism of PANI@N‐WHL‐AC

**FIGURE 6:**
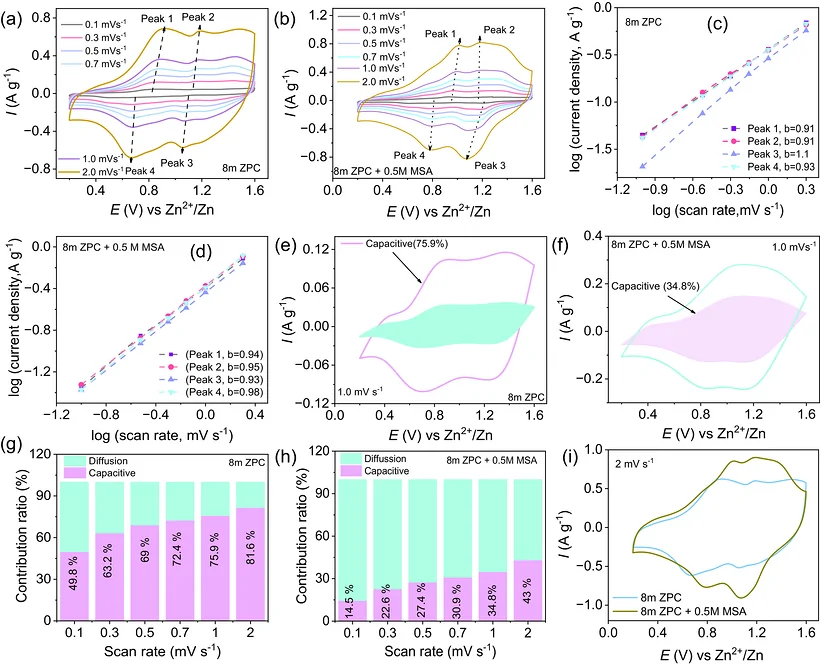
CV curves of PANI@N‐WHL‐AC in (a) 8 m ZPC and (b) ZPC + 0.5 M MSA at scan rates 0.1, 0.3, 0.5,0.7,1.0 and 2.0 mVs^−1^. Dunn's plot of PANI@N‐WHL‐AC (c) in 8 m ZPC_,_ and (d) 8 m ZPC + 0.5 M MSA. Capacitive contribution of PANI@N‐WHL‐AC in total capacity at 1 mV s^− 1^ in (e) 8 m ZPC, and (f) 8 m ZPC + 0.5 M MSA. Histogram of charge storage contribution of PANI@N‐WHL‐AC for diffusion‐controlled and capacitance‐controlled processes in (g) 8 m ZPC, and (h) 8 m ZPC + 0.5 M MSA. (i) overlaid CV of PANI@N‐WHL‐AC in the two electrolytes at 2 mVs^−1^.

To gain insight into the charge storage mechanism of PANI@N‐WHL‐AC, CV responses at low scan rates ranging from 0.1 to 2 mV s^−1^ in 8 m ZPC and 8 m ZPC + 0.5 M MSA (Figure 6a,b) were analyzed using the power law relationship (equation 1). To elucidate the reaction kinetics in both electrolytes, the *b*‐values were extracted from the relationship between peak current and scan rates according to the relation:

(1) $$i_{p}=av^{b}$$

where a *b*‐value close to 0.5 indicates a diffusion‐controlled process, while a *b*‐value approaching 1.0 suggests a capacitive or pseudocapacitive (surface‐controlled) mechanism. As shown in Figure 6c,d, the extracted *b*‐values corresponding to four representative anodic and cathodic peaks (Peak 1 to Peak 4) were 0.91, 0.91, 1.10, and 0.93 in 8 m ZPC, and 0.94, 0.95, 0.93, and 0.98 in 8 m ZPC + 0.5 M MSA, respectively. In both electrolytes, the *b*‐values approach unity, indicating that the overall charge storage process is predominantly governed by capacitive or pseudocapacitive contributions.

In contrast to peak‐based *b*‐value analysis, capacitive and diffusion‐controlled contributions were further quantified by Dunn's method according to the relationship (Equation 2):

(2) $$i\;\left(V\right)=k_{1}\;v+k_{2}v^{1/2}$$

here, k_1_ *v* represents the capacitive (surface‐controlled) current contribution and $k_{2}v^{1/2}$ corresponds to the diffusion‐controlled contribution. Figure 6e—h illustrates the separation of current contributions at different scan rates. As summarized in Figure 6g,h, the capacitive contribution in 8 m ZPC increased progressively from 49.8% to 81.8% as the scan rate increased from 0.1 to 2.0 mV s^−1^, confirming that surface‐controlled processes dominate charge storage in this electrolyte. However, upon the addition of 0.5 M MSA (Figure 6f,h), the capacitive contribution was significantly lower, increasing only from 14.5% to 43.1% over the same scan rate range. This result indicates a pronounced shift toward diffusion‐controlled behavior in the MSA‐containing electrolyte. The increased diffusion contribution could be attributed to Zn^2+^ intercalation and/or enhanced proton‐coupled redox reactions facilitated by the PANI@N‐WHL‐AC framework. Although *b*‐value and capacitive contribution analyses suggest different dominant mechanisms, this apparent discrepancy can be rationalized by considering the physical basis of each method. The *b*‐value analysis is derived from peak currents at specific peak potentials and primarily reflects fast surface redox processes, such as rapid H^+^ adsorption/desorption at or near the electrode interface. This method does not fully capture slower bulk ion transport processes, including Zn^2+^ intercalation, and therefore tends to emphasize surface‐controlled behavior. In contrast, the capacitive contribution analysis evaluates the entire CV profile across the full potential window (e.g., 0.2‐1.6 V), incorporating current responses at all potential ranges. This comprehensive approach accounts for both fast interfacial reactions and slower bulk diffusion processes. Consequently, the lower capacitive contribution and higher diffusion‐controlled contribution observed in the MSA‐containing electrolyte reflect the operation of a dual‐ion (Zn^2+^/H^+^) charge storage mechanism in the PANI@N‐WHL‐AC electrode. The proposed dual‐ion storage mechanism is further supported by the enlarged enclosed CV area observed in 8 m ZPC + 0.5 M MSA (Figure 6i), which indicates the combined contribution of Zn^2+^ and H^+^ to the overall charge storage process. Similarly, the Nyquist plot (Figure S9) exhibits a pronounced reduction in charge transfer resistance (*R*_ct_) and suppressed Warburg impedance in the 8 m ZPC + 0.5 M MSA implying improved interfacial kinetics with fast H^+^ and favorable Zn^2+^ co‐intercalation. In this system, the redox activity of PANI involves reversible transitions among the leucoemeraldine (fully reduced), emeraldine base (partially oxidized), and pernigraniline (fully oxidized) states during the charge–discharge process. The emeraldine base form contains both amine (─NH─) and imine (─C═N─) functional groups. Upon protonation by H^+^ ions, the imine sites convert into the emeraldine salt form, generating mobile charge carriers in the form of polarons and bipolarons. These charge carriers facilitate rapid electron transport and enable fast pseudocapacitive charge storage at or near the electrode surface. At deeper discharge states, Zn^2+^ intercalation into the PANI matrix occurs, contributing an additional diffusion‐controlled, battery‐type component to the overall charge storage mechanism. Therefore, the coexistence of fast proton‐coupled surface redox reactions and slower Zn^2+^ intercalation processes confirms the hybrid nature of charge storage in the PANI@N‐WHL‐AC electrode [92, 93, 94, 102, 103, 104].

## 3 Conclusions

In this work, a synergistic PANI@N‐WHL‐AC composite cathode was developed as a high‐performance platform for aqueous ZIBs through rational electrolyte engineering. The incorporation of MSA into the concentrated 8 m ZPC electrolyte significantly improved ionic conductivity, reaction kinetics, and electrode/electrolyte interfacial stability. This electrolyte optimization enabled a regulated dual‐ion (Zn^2+^/H^+^) storage mechanism and enhanced Zn plating/stripping reversibility. The complementary pseudocapacitive behavior of PANI and the electrical double‐layer contribution of the water‐hyacinth‐derived activated carbon framework synergistically improve electrochemical performance, delivering a specific capacity of ≈105.6 mAh g^−1^ at 1.0 A g^−1^, an energy density of ≈101.5 Wh kg^−1^, a power density of ≈692.8 W kg^−1^, and stable cycling over 2500 cycles with ≈ 80% capacity retention. Overall, this work highlights the critical role of electrolyte design in controlling water structure, Zn^2+^ solvation, and charge‐storage pathways, and demonstrates a viable strategy for developing high energy, long‐life, and sustainable aqueous ZIB systems.

## 4 Experimental Section

### 4.1 Materials and Chemicals

Water hyacinth leaves derived activated carbon (WHL‐AC), Aniline (C_6_H_5_NH_2_, 99.5%, Riedelde Haen), Potassium hydroxide (KOH, 98%, Sigma‐Aldrich), zinc chloride (ZnCl_2_, 98%, Fluka, Germany), ammonium persulfate ((NH_4_)_2_S_2_O_8_, 99%, BDH, England) poly(vinylidene fluoride) (PVDF, Sigma–Aldrich), *N*‐methyl‐2‐pyrrolidone (NMP, Sigma‐Aldrich), Zinc perchlorate hexahydrate(Zn(ClO_4_)_2_.6H_2_O), Methanesulfonic acid(≥ 99%, Sigma Aldrich), Zn foil(thickness:50 micrometer, goodfellow), graphite foil(thickness:150 micrometer, SIGRATHERM), Whatman glass fiber filter (GF/A).

### 4.2 Synthesis of PANI@N‐WHL‐AC Composite

**SCHEME 1:**
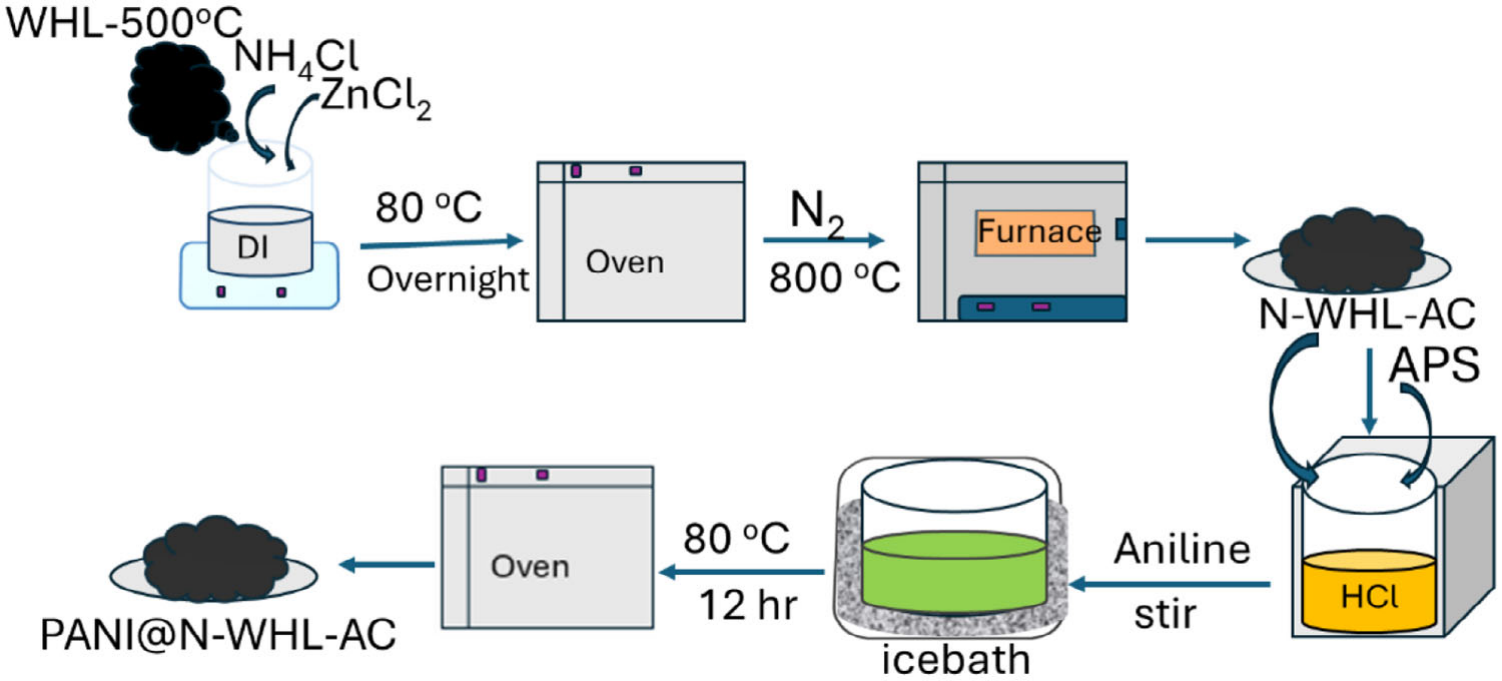
The schematic representation of in situ polymerization of aniline on N‐doped water hyacinth‐derived activated carbon.

WHL‐AC was prepared following the procedure described in the literature [105]: briefly, water hyacinth leaf powder (5 g, 200 µm) was thermally annealed in an atmosphere furnace at 500°C under N_2_ conditions for 1 h. N‐WHL‐AC was prepared by following the procedure described in the literature [49] with some modifications. Briefly, 4 g of ZnCl_2_ and 1 g of NH_4_Cl were added to 50 mL of deionized water to form a solution, followed by the addition of 2 g of WHL carbonized at 500^°^C. The mixture was then stirred at room temperature for 8 h and dried in an oven at 80^°^C overnight. Afterward, the mixture was placed in a furnace filled with flowing N_2_ gas and annealed at 800^°^C for 1 h with a heating rate of 10^°^C min^−1^. Finally, the carbon product was collected after being thoroughly washed with 2 M HCl and distilled water and dried at 80^°^C to remove any inorganic impurities. PANI was synthesized by the in situ chemical oxidative polymerization technique using aniline as the monomer, ammonium persulfate (APS as an oxidant, and HCl as a dopant, as supported by reference [51]. A PANI@N‐WHL‐AC composite was synthesized by the in situ chemical oxidative polymerization technique, as reported in the reference [51] with some modifications. In brief, 2.5 g of APS was added to 50 mL of 1 M HCl, and 100 mg of N‐WHL‐AC was taken in 50 mL of 1 M HCl, followed by sonication for 2 h. Afterward, the resulting solution was kept in an ice bath, and 1 mL of aniline was added to it dropwise with continuous stirring for 24 h. Finally, the resulting PANI@N‐WHL‐AC was washed with DI water several times, filtered, and oven dried at 80^°^C for 12 h. The overall synthesis route is represented in Scheme 1.

### 4.3 Electrode Preparation and Cell Assembly

The slurry of PANI@N‐WHL‐AC was prepared in an 80:10:10 ratio for PAN@N‐WHL‐AC, conductive carbon black (Super P), and PVDF as binder, respectively, in NMP solvent. The slurry was stirred until it became homogeneous and coated with a bar coater on graphite foil. Then the coated graphite foil was dried in an oven for 12 h at 60 ^°^C. A similar procedure was followed for N‐WHL‐AC and PANI for comparison. The coin cell type‐ CR2032 was used for measuring the electrochemical performance of the battery. Asymmetric cells were assembled open‐air from the PANI or N‐WHL‐AC or PANI@N‐WHL‐AC and zinc foil as the positive and negative electrodes, glass fiber type A (GF/A) as separator in 8 m ZPC and 8 m ZPC + 0.5 M MSA for CV and GCD study each material, cycling stability, specific energy density and specific power density estimation and Energy storage mechanism of the material under study.

For device measurements, the cells were charged at various currents ranging from 0.05 to 1.0 A g^−1^ within a potential window of 0.2–1.6 V. The chronopotentiometry (CP) technique was employed for galvanostatic charge–discharge (GCD) testing and for determining stored capacity. Specific energy (*E*) and specific power (*P*) were calculated using Equations (3) and (4), where *m* is the mass of active materials, *t* is the discharge time, *i* is the discharge current, and V is the potential.

(3) $$E=\frac{1}{m}\;\int_{0}^{t}iVdt\;$$

(4) $$P=\frac{E}{t}\;$$

### 4.4 Electrolyte Characterization

The prepared electrolytes were characterized extensively to get insights into the change in solvation structure of Zn^2+^ ions. To understand, Attenuated Total Reflectance Fourier‐Transform Infrared spectroscopy (ATR‐FTIR) of the 2 m ZPC, 2 m ZPC +0.5 M MSA, 8 m ZPC, and 8 m ZPC +0.5 M MSA electrolytes were collected using an ATR‐FTIR spectrometer (Vertex 70, Bruker) with a range from 4000 to 450 cm^−1^ under ambient conditions. The impedance measurements were carried out using an impedance spectrometer (Alpha high‐resolution dielectric analyzer, Novocontrol Technologies GmbH, Hundsangen, Germany) with the Cavity BDS1308. An AC voltage of 10 mV was applied while sweeping the frequency. The bulk ionic conductivity was then estimated by using the equation: $\sigma =\frac{d}{R\;xA}\;$ where d, A, and R are the distance between the electrodes, area of cross section, and resistance, respectively.

Before device electrochemical characterization, the CR2032 Zn||Zn symmetric cell batteries were assembled in the open‐air using two Zn foils (diameter 16 mm), and GF/A(diameter 20 mm) to study zinc plating and stripping behavior in 8m ZPC and 8 m ZPC +0.5 M MSA. Similarly, Zn||Zn symmetric cell batteries were assembled to investigate the Zn deposition in 8 m ZPC +0.5 M MSA after 10 and 50 cycles using two different cells. Then the SEM and XRD were employed for surface characterization of Zn foil for Zn deposition after 10 and 50 cycles, and after cycling. For the corrosion formation test, the pristine Zn foil was soaked in 8m ZPC, and 8m ZPC + 0.5 M MSA for 72 h and electrodes were characterized by XRD to observe any formation of any side product. The three‐electrode system constructed from GCE, Ag/AgCl, and Pt mesh as working, reference, and counter electrodes, respectively were used to measure the ESW of the 8 m ZPC and 8 m ZPC +0.5 M MSA. The CR2032 Zn||Ti asymmetric cell was assembled in the same way as the Zn||Zn symmetric cell, except that the positive electrode was replaced by a Ti mesh to study coulombic efficiency in the 8 m ZPC and 8 m ZPC + 0.5 M MSA.

## Conflicts of Interest

The authors declare no conflicts of interest.

## Supporting information




**Supporting File**: smll74563‐sup‐0001‐SuppMat.docx.

## Data Availability

The data that support the findings of this study are available from the corresponding author upon request.
