# Supplementary material for: Boosting Performance of PANI@N‐WHL‐AC by a Modified Water‐in‐Salt‐Electrolyte Strategy for Zinc‐Ion Batteries
Source: Small. 2026 Jul 14;22(50):e74563. doi: 10.1002/smll.74563 (PMC13548951; doi:10.1002/smll.74563)
Supplement: Supplementary file 1 [file SMLL-22-e74563-s001.docx]

**Boosting Performance of PANI@N-WHL-AC by a Modified Water-in-Salt-Electrolyte Strategy for Zinc-ion Batteries**

Olbana Itana,*^a,b,c^* Asmamew Taye,*^d^* Xinwen Peng,*^e^* Varsha Joseph,*^a,f^* Amare Aregahegn,*^b^* Reverant Crispin,*^a,f^*, Shimelis Admassie,*^g^* Ziyauddin Khan*^a,f^*

*^a^*Laboratory of Organic Electronics, Department of Science and Technology (ITN), Linköping University, Norrköping SE-60174, Sweden

*^b^*Center of Materials Science and Engineering, Addis Ababa University, PO BOX 1176, Addis Ababa, Ethiopia

*^c^*Department of Chemistry, College of Natural and Computational Sciences, Wallaga University, Nekemte 395, Ethiopia

*^d^*Department of Chemistry, Asosa University, PO BOX 18, Asosa, Ethiopia

*^e^*State Key Laboratory of Pulp and Paper Engineering, South China University of Technology,

Guangzhou 510641, China

*^f^*Wallenberg Wood Science Center, Linköping University, Norrköping SE-601 74, Sweden

*^g^*Department of Chemistry, Addis Ababa University, PO Box 1176, Addis Ababa, Ethiopia

E-mail: [ziyauddin.khan@liu.se](mailto:ziyauddin.khan@liu.se) (Ziyauddin Khan); [reverant.crispin@liu.se](mailto:reverant.crispin@liu.se) (Reverant Crispin); [shimelis.admassie@aau.edu.et](mailto:shimelis.admassie@aau.edu.et) (Shimelis Admassie)

**Figure S1:** XPS survey spectra of N-WHL-AC, PANI, and PANI@N-WHL-AC

**Table S1.** Elemental composition of N-WHL-AC, PANI, and PANI@N-WHL-AC.

| Elemental composition (%) | | | |
| --- | --- | --- | --- |
| The constituent elements | C | O | N |
| N-WHL-AC | 81.29 | 11.70 | 6.68 |
| PANI | 78.20 | 8.5713 | 13.05 |
| PANI@N-WHL-AC | 75.88 | 11.79 | 12.22 |

**Figure S2:** (a,b) ATR-FTIR spectra of various electrolytes in lower wavenumber region.

**Electrochemical plating/stripping kinetics using CV**

**Figure S3:** CV of zinc plating and stripping at various scan rates from 1-10 mV s^-1^ in (a) pristine 8 m ZPC electrolyte and (b) 8 m ZPC + 0.5 M MSA. All potentials are referenced to Zn^2+^/Zn.

**Post-cycling SEM analysis of Zn foil in 8m ZPC and 8 m ZPC + 0.5 M MSA**

Figure S4 shows SEM images of Zn electrodes recovered from Zn||Zn symmetric cells after galvanostatic plating/stripping in 8 m ZPC and 8 m ZPC + 0.5 M MSA electrolytes. The Zn electrode cycled in pristine 8 m ZPC (Figure S4a,b) exhibits a heterogeneous surface morphology with visible cracks and irregular surface features, indicating significant surface reconstruction during repeated Zn plating/stripping. In contrast, the Zn electrode cycled in 8 m ZPC + 0.5 M MSA (Figure S4 c,d) displays a more compact and uniformly distributed platelet-like morphology. The clear difference in surface morphology suggests that MSA modifies Zn deposition behavior and contributes to a more stable Zn/electrolyte interface during prolonged cycling.

**Figure S4:** SEM images of Zn electrodes recovered from Zn||Zn symmetric cells after galvanostatic cycling in (a,b) 8 m ZPC and (c,d) 8 m ZPC + 0.5 M MSA electrolytes.

**Corrosion test of Zn foil dipped in electrolyte**

In addition to post-cycling SEM analysis, the chemical stability of Zn in the two electrolytes was evaluated by immersing Zn foils in 8 m ZPC and 8 m ZPC + 0.5 M MSA for 72 h, followed by XRD characterization (Figure S5). The XRD patterns mainly show characteristic peaks of metallic Zn indicating that prolonged electrolyte exposure does not induce significant crystalline corrosion products. These results provide complementary evidence that the MSA-containing electrolyte better preserves the structural integrity.

**Figure S5:** XRD patterns of Zn foil dipped in 8 m ZPC electrolytes with/without MSA.

**Figure S6:** Coulombic efficiency of Zn plating/stripping in Ti||Zn asymmetric cells cycled in 8 m ZPC and 8 m ZPC + 0.5 M MSA electrolytes for 50 cycles at an areal capacity 1mAh cm^-2^.

**Figure S7:** CV curves of N-WHL-AC (a) and PANI (c) at different scan rates and GCD profiles of N-WHL-AC (b) and PANI (d) at different current densities in 8 m ZPC + 0.5 M MSA electrolyte. Each current density in the GCD rate test was applied for two consecutive cycles.

**Figure S8:** Self-discharge behavior of Zn||PANI@N-WHL-AC full cells. (a) Open-circuit voltage (OCV) decays after charging to 1.6 V followed by 120 h rest. (b) Discharge profile after OCV aging, showing the remaining capacity.

**Table S2:** Self-discharge comparison of PANI and other cathode materials in Zn-based systems.

| Cathode | Electrolyte | Current rate (A/g) | Charge potential (V) | Rest period (h) | End potential after OCV (V) | Capacity retention after OCV (%) | Ref |
| --- | --- | --- | --- | --- | --- | --- | --- |
| PANI | 2M ZnSO_4_ |  | 1.5 | 24 | 0.98 | 24 | ^[1]^ |
| PANI | Inorganic silica gel electrolyte |  | 1.5 | 24 | 1.2 | 90 | ^[1]^ |
| Mn ion-doped polyaniline(PAM) | 2 M ZnSO_4_ | 1 | 1.8 V | 24 |  | 94.3 | ^[2]^ |
| PANI | 2 wt% carboxybetaine oligomeric additive in ZnSO_4_ |  | 1.5 V | 24 | 0.5 | 96.2 | ^[3]^ |
| Anthraquinone- diacetylene/PANI | 2M Zn(ClO_4_)_2_.6H_2_O | 0.1 | 1.8 | 350 | 1.04 |  | ^[4]^ |
| Black phosphorus | 21 M LiTFSI+1 M Zn(CF_3_SO_3_)_2_ | 0.5 | 2.2 | 300 | 1.47 | 76.16 | ^[5]^ |
| N-doped Ti_3_C_2_ | 1M ZnSO_4_ | 0.1 | 1.2 | 240 | 0.903 |  | ^[6]^ |
| Hierarchical porous carbon fiber | 2M ZnSO_4_ | 0.1 | 1.8 | 24 | 1.42 |  | ^[7]^ |
| PANI | 1-ethyl-3-methylimidazoliumiodide+ 2M ZnSO_4_.7H_2_O |  | 1.5 | 48 | ~1.3*^a^* | 92.7 | ^[8]^ |
| TiN | ZnSO_4_ |  | 1.9 | 500 | 1.22 | 83.92 | ^[9]^ |
| TiN | ZnSO_4_ |  | 1.9 | 500 | >1*^a^* | 76.7 | ^[9]^ |
| PANI@N-WHL-AC | 8 m ZPC+0.5M MSA | 0.5 | 1.6 | 120 | 1.1 | 53.6 | This work |

^a^Estimated end potential extracted from reported data..

**Figure S9:** Nyquist plots of Zn||PANI@WHL-AC in 8 m ZPC and 8 m ZPC + 0.5 M ZPC electrolytes.

**References**

1.	Zheng, R., Wang, B., Liu, Y., Zhang, J., Hou, C., Zhang, Q., Li, K., Li, Y., and Wang, H. (2025) Zinc-ion coordinated inorganic silicone quasi-solid electrolyte for low self-discharge and stable zinc metal batteries. *Electrochimica Acta*, **530**, 146409.

2.	Gong, S., Zhu, M., Zhou, Y., Li, R., Zhang, J., Jia, X., Chao, D., and Wang, C. (2024) An interactive dual energy storage mechanism boosts high-performance aqueous zinc-ion batteries. *Chem. Sci.*, **15** (47), 19870–19885.

3.	Deng, Y., Xu, S., Peng, Z., Fei, L., and Wang, T. (2026) Unveiling bulk-to-interface electrolyte regulation for ultralong-life Zn-ion batteries. *Chem. Sci.*, **17** (13), 6662–6676.

4.	Samage, A., Slavsky, E., and Jelinek, R. (2026) High-performance polydiacetylene organic cathode for zinc-ion batteries. *J. Mater. Chem. A*.

5.	Huang, Z., Chen, A., Mo, F., Liang, G., Li, X., Yang, Q., Guo, Y., Chen, Z., Li, Q., Dong, B., and Zhi, C. (2020) Phosphorene as Cathode Material for High-Voltage, Anti-Self-Discharge Zinc Ion Hybrid Capacitors. *Adv. Energy Mater.*, **10** (24), 2001024.

6.	Jin, Y., Ao, H., Qi, K., Zhang, X., Liu, M., Zhou, T., Wang, S., Xia, G., and Zhu, Y. (2021) A High-rate, Long Life, and Anti-self-discharge Aqueous N-doped Ti3C2/Zn Hybrid Capacitor. *Mater. Today Energy*, **19**, 100598.

7.	Li, Y., Yang, W., Yang, W., Wang, Z., Rong, J., Wang, G., Xu, C., Kang, F., and Dong, L. (2021) Towards High-Energy and Anti-Self-Discharge Zn-Ion Hybrid Supercapacitors with New Understanding of the Electrochemistry. *Nano-Micro Lett.*, **13** (1), 95.

8.	Wu, Y., Wen, L., Wei, S., Shi, J., Zhou, Q., Zhou, Q., Jiang, W., Wu, X., Chimtali, P.J., Chi, Q., Wang, C., and Song, L. (2026) Interfacial chemistry modulation of Zn anodes via an EMImI ionic liquid additive for stable aqueous zinc-ion batteries. *Nanoscale*, **18** (4), 2202–2214.

9.	Huang, Z., Wang, T., Song, H., Li, X., Liang, G., Wang, D., Yang, Q., Chen, Z., Ma, L., Liu, Z., Gao, B., Fan, J., and Zhi, C. (2021) Effects of Anion Carriers on Capacitance and Self-Discharge Behaviors of Zinc Ion Capacitors. *Angew. Chem. Int. Ed.*, **60** (2), 1011–1021.
